# Evaluating the Anchorflow Suture Technique Versus Conventional Continuous Suturing in Vaginal Cuff Closure During TLH: A Multicenter Retrospective Analysis

**DOI:** 10.3390/medicina61050813

**Published:** 2025-04-28

**Authors:** Gizem Berfin Uluutku Bulutlar, Gizem Boz İzceyhan, Eralp Bulutlar, Fisun Vural

**Affiliations:** 1 Haydarpaşa Numune Eğitim ve Araştırma Hastanesi, Istanbul 34668, Turkey; fisunvural@yahoo.com.tr; 2 Zeynep Kamil Hospital, Istanbul 34668, Turkey; gizemboz@hotmail.com (G.B.İ.); eralpbulutlar@hotmail.com (E.B.)

**Keywords:** anchorflow suture, suture techniques, total laparoscopic hysterectomy, continue suture

## Abstract

*Background and Objectives*: Total laparoscopic hysterectomy (TLH) is a commonly performed gynecological procedure. Vaginal cuff closure significantly impacts operative time and outcomes. This study evaluates the newly developed Anchorflow Suture (AFS) technique compared to conventional continuous suturing in terms of efficiency and safety. *Materials and Methods*: A multicenter retrospective cohort study was conducted at two tertiary referral hospitals involving 208 women who underwent TLH for benign indications. Women were divided into two groups based on vaginal cuff closure technique: AFS and continuous suturing. Demographic characteristics, surgical parameters, and postoperative outcomes were analyzed using appropriate statistical tests, with a significance level of *p* < 0.05. *Results*: No significant differences were found between groups in age, BMI (body mass index), gravida, parity, or menopausal status. Vaginal cuff closure time was significantly shorter with AFS (10.26 ± 2.3 min) compared to continuous suturing (13.36 ± 2.8 min, *p* < 0.001). Operative time was shorter in the AFS group, though not statistically significant (*p* = 0.15). Both techniques demonstrated similar safety profiles, with no cases of vaginal cuff dehiscence and comparable rates of granulation tissue formation, bleeding, and urinary incontinence. The AFS group showed a slightly lower incidence of postoperative bleeding (five cases vs. three cases). *Conclusions*: The AFS technique significantly reduces vaginal cuff closure time and demonstrates a comparable safety profile to continuous suturing. This method enhances surgical efficiency without increasing complications. Further prospective studies are needed to evaluate its long-term effects on pelvic floor integrity, sexual function, and surgeon proficiency.

## 1. Introduction

Total hysterectomy is one of the most frequently performed gynecological surgeries worldwide, with a growing preference for laparoscopic approaches, particularly in benign cases [1]. The utilization of minimally invasive surgical techniques has been increasingly adopted worldwide due to advantages such as faster mobilization, earlier return to daily activities, and shorter hospital stays. Consequently, the proportion of total laparoscopic hysterectomy (TLH) among hysterectomy procedures is rising [2,3,4]. The American Congress of Obstetricians and Gynecologists (ACOG) strongly recommends a minimally invasive approach to maximize patient benefits [5].

In the course of TLH, cuff suturing is a critical step that significantly impacts surgical duration. Various techniques and suture materials have been explored to optimize this process. The application of different suturing techniques aims to not only reduce operative time but also minimize postoperative complications such as vaginal cuff dehiscence(VCD) [6,7,8].

Initially, the intracorporeal suturing technique required advanced surgical skills and prolonged operative time. Therefore, transvaginal cuff closure (extracorporeal suturing) has been investigated as an alternative method. Nevertheless, studies have suggested that this technique may be associated with a higher incidence of vaginal dehiscence and increased complication rates [9,10].

When reviewing meta-analyses evaluating the effects of barbed and conventional sutures on vaginal cuff closure in TLH, no statistically significant difference has been observed in the incidence of VCD [9].

Currently, there is no standardized protocol regarding the suture material or technique used for vaginal cuff closure [1]. The aim of this study is to compare our newly developed intracorporeal Anchorflow suture technique (AFS) with the continuous suturing technique for vaginal cuff closure and to evaluate whether this technique offers similar advantages in terms of reducing operative time and enhancing resistance to tissue tension.

## 2. Materials and Methods

This multicenter retrospective cohort study was designed to evaluate the outcomes of different suturing techniques in TLH. Ethical approval was obtained from the Haydarpaşa Numune Research and Training Hospital Clinical Research Ethics Committee with Decision No. HNEAH-BAEK 2024/166. A total of 208 patients, aged 40–65 years, who underwent TLH for benign indications between 1 January 2022, and 1 December 2024, were included in the study. The procedures were performed by surgical teams with similar levels of experience at Haydarpaşa Numune Research and Training Hospital and Zeynep Kamil Women and Children’s Diseases Training and Research Hospital.

**Figure 1:**
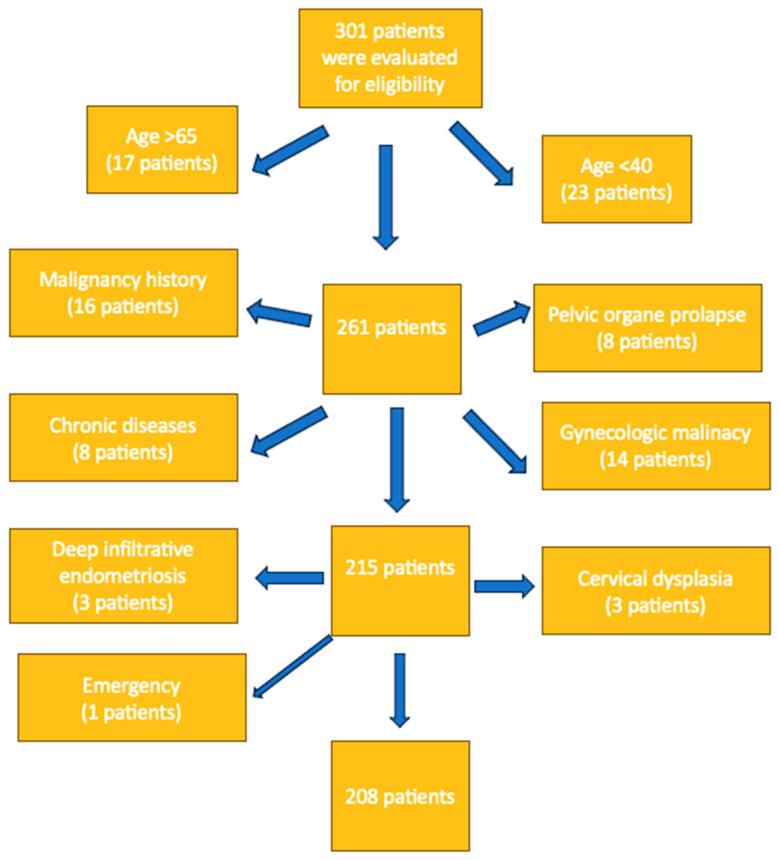
Eligibility criteria and exclusions.

Inclusion and Exclusion Criteria: Women aged between 40 and 65 years who underwent total laparoscopic hysterectomy (TLH) for benign gynecological conditions were included in this study. The age range was selected to target a relatively homogeneous peri- and post-menopausal population, in which benign uterine pathologies are most prevalent and wound-healing characteristics are more stable [11]. The exclusion criteria were designed to minimize confounding variables that could influence wound healing, surgical complexity, or postoperative outcomes. Women were excluded if they had diabetes mellitus, were under immunosuppressive treatment, had a history of malignancy or tubo-ovarian abscess, or underwent surgery for deep infiltrative endometriosis. Particularly, patients with malignancy were excluded because oncologic surgical protocols involve more extensive tissue dissection and possible adjuvant therapies, which could affect surgical time, healing, and complication rates [12]. Additional exclusions included cases performed in emergency settings, as well as women under 40 or over 65 years of age. These exclusions ensured better standardization of the patient population and allowed for a more controlled comparison between the two suturing techniques. All patients were classified as ASA I or ASA II according to the American Society of Anesthesiologists Physical Status Classification; no patients with ASA III or higher were included in the study (Figure 1).

Data retrieved from the hospital records included patient age, gravida, parity, body mass index (BMI), menopausal status, vaginal cuff closure techniques, time to close vaginal cuff(minute), length of surgery(minute), changes in preoperative and postoperative hematocrit levels, emergency situations, postoperative hospital stay time(day), 3-week postoperative findings such as dehiscence, infection, bleeding, and 6-week postoperative findings dehiscence, vault granulation tissue formation, and uriner incontinance. No patients required intraoperative blood transfusion in either group. Infection was defined as the surgical site infection (SSI) based on clinical findings such as localized pain, redness, purulent discharge, or fever in accordance with CDC criteria [13].

Patients underwent total laparoscopic hysterectomy with vaginal cuff closure using polyglactin 910 (coated Vicryl suture; Ethicon/Johnson & Johnson; New Brunswick, NJ, USA). As all surgical teams were proficient in both methods, the choice between intracorporeal continuous suturing and the Anchorflow suture (AFS) technique was made intraoperatively, based on surgeon preference, as part of a retrospectively evaluated, non-randomized design.

All patients underwent standard preoperative preparation procedures. Prophylactic antibiotics (Cefazolin 2 g intravenously) were administered 30 min before the incision, along with compression stockings. Following general anesthesia and endotracheal intubation, the patients were positioned in the Trendelenburg position with lithotomy. After appropriate field preparation and sterile draping, a 14-Fr Foley catheter was inserted into the bladder. The procedure was performed with patients in the lithotomy position and started with a uterine manipulator; four trocars were placed in total: one umbilical of 10 mm and three accessories of 5 mm. A 30–angled 10 mm endoscope (Karl Storz SE & Co. KG, Tuttlingen, Germany), a grasper (ENDOPATHTM Grasper 5 mm, Ethicon, Inc., Somerville, NJ, USA), and bipolar forceps (LigaSure™ Medtronic, Minneapolis, MN, USA) were used to coagulate vascular ligaments and pedicles, and monopolar energy (60 W) was used to perform the colpotomy with the monopolar hook at the height of the uterine manipulator cup. Two distinct intracorporeal suturing techniques were employed for the closure, each differing in approach, yet both performed entirely within the abdominal cavity.

**Figure 2:**
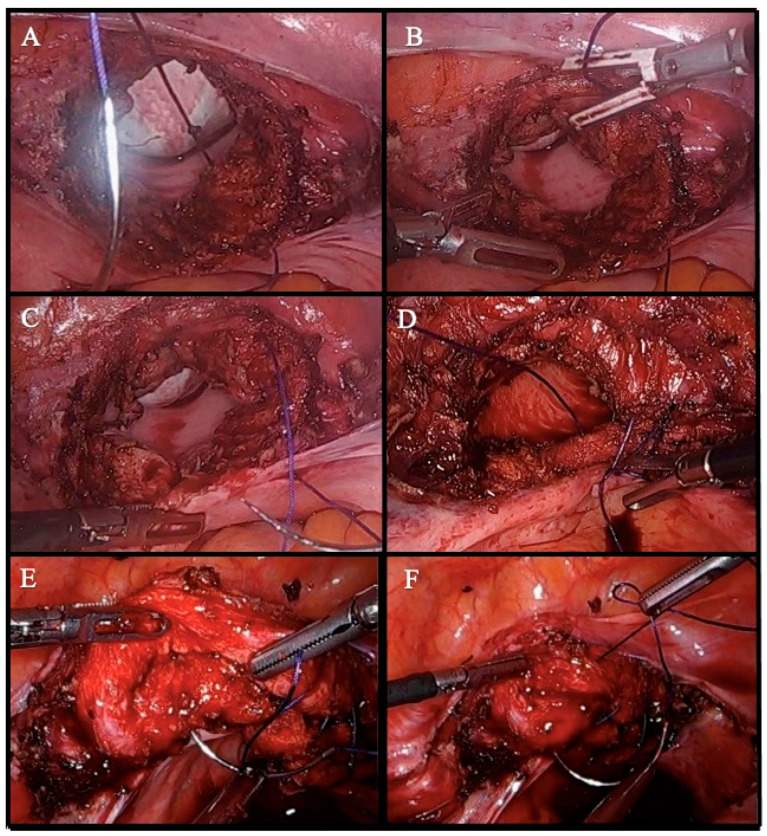
Step-by-step illustration of vaginal cuff closure using a continue suture technique. (**A**) The suturing begins from the lower margin of one end of the vaginal cuff. (**B**) The needle is passed through the upper margin of the same corner to prepare for knot tying. (**C**) The first knot is secured at the corner, anchoring one end of the cuff. (**D**) The running suture is continued toward the opposite end at regular intervals, ensuring a consistent suture line. (**E**) As the suture approaches the opposite end, the contralateral uterosacral ligaments are included. The cuff’s symmetry and completeness are evaluated before the final pass, and additional stitches are placed if necessary. (**F**) The final knot is tied, completing the closure. The integrity of the closure is checked, and any bleeding or gaps along the suture line are addressed.

Continue suture group: The suturing process starts with a knot at one end of the vaginal cuff, ensuring that the uterosacral ligaments are included. The suture is then continued in a running fashion to the opposite end, where a final knot is placed for secure closure (Figure 2).

Anchorflow suture group (AFS): In this technique, vaginal cuff closure begins with the placement of a single 0-Vicryl suture through the full thickness of the vaginal cuff at the midline, incorporating both anterior and posterior layers. A secure intracorporeal knot is tied to fix the suture. Following this, gentle upward traction is applied to the suture, allowing the bladder and bowel to retract and enhancing visualization of the operative field.

The needle is then advanced laterally from the midline toward the right vaginal cuff angle, with each pass incorporating the full thickness of the vaginal mucosa and the uterosacral ligament at its insertion to provide apical support. Upon reaching the lateral edge, the suture is kept under tension, and the same suture is redirected back through the midline in a mirrored fashion toward the left vaginal cuff angle, repeating the same tissue incorporation pattern.

**Figure 3:**
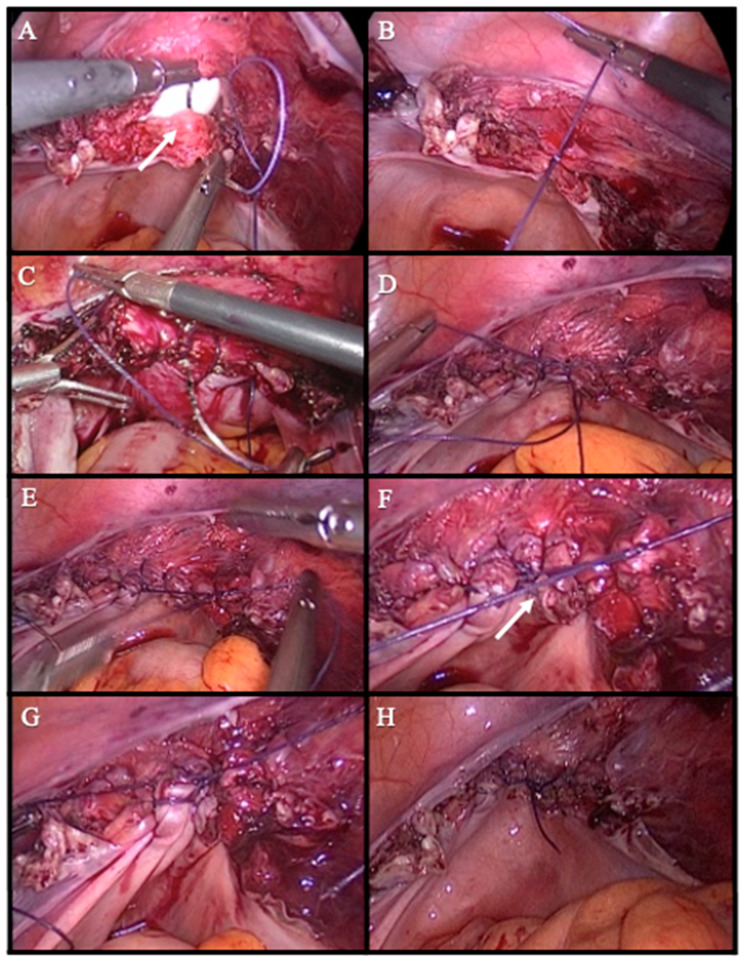
Step-by-step illustration of vaginal cuff closure using anchorflow suture technique. (**A**) A single Vicryl suture is placed at the midline of the vaginal cuff, incorporating both the anterior and posterior layers. white arrow indicates the midline of the vaginal cuff where the initial suture is placed (**B**) The first knot is tied to secure and anchor the initial suture. (**C**) The suture is advanced from the midline toward the right vaginal cuff angle, incorporating the full thickness of the vaginal mucosa and the uterosacral ligament with each pass. (**D**) The same suture line is continued symmetrically from the midline toward the left vaginal cuff angle. (**E**) The free end of the suture is approximated to the initial knot at the midline. (**F**) A final intracorporeal knot is tied at the midline to complete the closure of the vaginal cuff. white arrow shows the point where the final suture is tied (**G**) Hemostasis is confirmed, and the suture line is inspected for any gaps before concluding the suturing. (**H**) This technique not only ensures a secure closure but also provides bilateral uterosacral ligament suspension, enhancing pelvic support and reducing the risk of cuff prolapse.

At the end of the second arm of the suture, a final intracorporeal knot is tied by approximating the free end of the suture with the original anchoring knot at the midline. This technique not only ensures firm closure of the vaginal cuff but also results in bilateral uterosacral ligament suspension, contributing to pelvic support and minimizing the risk of cuff prolapse. The technique was performed entirely intracorporeally using standard laparoscopic needle drivers (Figure 3).

For cuff closure with the Vicryl suture, we used the 0 Vicryl suture with the vaginal cuff closure proceeding. Finally, the hemostasis and ureteral trajectories were checked, carbon dioxide was evacuated, the trocars were removed, and the skin was closed.

All patients underwent postoperative evaluations on the 3rd and 6th weeks following the surgical procedure. These assessments were conducted in an outpatient setting by one of the operating surgeons, following a standardized follow-up protocol to ensure consistency across all cases [11]. All patients underwent a vaginal examination to check for vaginal cuff complications. All abnormal findings were recorded.

### Statistical Methods

Statistical analyses were performed using IBM SPSS Statistics for Windows, Version 23.0 (IBM Corp., Armonk, New York, NY, USA). Descriptive statistics were used to summarize the data: categorical variables were expressed as frequencies and percentages, and continuous variables were expressed as means with standard deviations, or medians with minimum and maximum values where appropriate. The Kolmogorov–Smirnov test was used to evaluate the normality of the distribution of continuous variables. For comparisons between two independent groups, the Student’s *t*-test was used for normally distributed variables, and the Mann–Whitney U test was used for non-normally distributed variables. The Chi-square test or Fisher’s exact test was applied for comparisons of categorical variables, depending on expected frequencies. A *p*-value of <0.05 was considered statistically significant, and results were presented with 95% confidence intervals (CIs) where applicable. An a priori power analysis was performed to determine the adequacy of the sample size. Based on a medium effect size (Cohen’s d = 0.5), a significance level of α = 0.05, and a desired statistical power of 0.80, the minimum required sample size was calculated to be approximately 75 patients per group. With 120 patients in the continuous suturing group and 88 in the AFS group, the actual sample size was deemed sufficient to detect statistically significant differences in the primary outcome (vaginal cuff closure time). To minimize the potential impact of confounding variables, strict exclusion criteria were applied. Patients with diabetes mellitus, immunosuppressant use, malignancy, tubo-ovarian abscess, or endometriosis were excluded due to their potential to influence wound healing and surgical outcomes. This strategy enhanced the internal validity of the comparisons made between the two suturing techniques. Although the study was adequately powered for the primary endpoint, the relatively small number of postoperative complications may have limited the statistical power for detecting significant differences in secondary outcomes, such as infection, bleeding, or granulation tissue formation. Further studies with larger cohorts are warranted to better evaluate these parameters.

## 3. Results

A total of 208 patients who underwent TLH with either continuous suturing or the newly developed AFS technique were included in this study. The demographic characteristics of the patients are summarized in Table 1.

There were no statistically significant differences between the two groups in terms of age, BMI, gravida, parity, preoperative and postoperative hematocrit levels, or menopausal status (*p* > 0.05).

When comparing the outcomes of both techniques, no statistically significant difference was observed between the groups in terms of mean hospital stay (continuous suturing: 2.02 ± 0.5 days vs. AFS: 1.99 ± 0.4 days, *p* = 0.84). Although the mean operative time was shorter in the AFS group compared to the continuous suturing group (96.13 ± 10.2 min vs. 103.98 ± 37.5 min), this difference did not reach statistical significance (*p* = 0.15). However, the vaginal cuff closure time was significantly shorter in the AFS group (10.26 ± 2.3 min vs. 13.36 ± 2.8 min, *p* < 0.001) (Table 2).

Additionally, no significant difference was found between the two groups in terms of preoperative and postoperative hematocrit level changes (continuous suturing: 4.73 ± 2.4 vs. AFS: 4.49 ± 2.2, *p* = 0.47), indicating similar blood loss in both techniques.

The postoperative complication rates are shown in Table 3.

There was no case of VCD in either postoperative period. The rate of postoperative bleeding was slightly lower in the AFS group (continuous suturing: 5 cases vs. AFS: 3 cases), but the sample size was too small to determine statistical significance. Vault granulation tissue formation at the 6-week follow-up was observed in one patient from each group. Additionally, urinary incontinence was reported in two patients from the continuous suturing group, while no cases were observed in the AFS group. There was only one case of emergency relaparotomy in the continuous suturing group, while none occurred in the AFS group.

Finally when we examine the results, we observe that AFS significantly reduces vaginal cuff closure time and has a similar safety profile to continue suture.

## 4. Discussion

This study compares the AFS technique, a newly developed method for vaginal cuff closure during TLH, with the conventional continuous suturing technique. Our findings indicate that while the AFS technique demonstrates a similar safety profile to the continuous suturing method, it significantly reduces vaginal cuff closure time.

The significantly shorter vaginal cuff closure time observed in the AFS group (10.26 ± 2.3 min vs. 13.36 ± 2.8 min, *p* < 0.001) can be attributed to several key factors. In the AFS technique, after the initial midline suture placement, traction is applied to the suture, allowing retraction of the bladder and bowel, which enhances surgical visualization. This improved visualization facilitates the simultaneous and more efficient suturing of both vaginal cuff layers in a single pass, thereby accelerating the closure process. Additionally, the enhanced surgical field and the ability to manipulate the suturing site more effectively enable the surgeon to perform more controlled and rapid suturing, ultimately reducing vaginal cuff closure time.

A comprehensive meta-analysis published in 2021 reported that the incidence of VCD after TLH ranges between 0.64% and 1.64% [9]. However, in our study, no cases of vaginal cuff dehiscence were observed in either group. This finding may be attributed to the exclusion of patients with factors that could impair wound healing, such as diabetes mellitus, advanced age, and malignancy, when comparing cuff-suturing techniques.

A review of the literature reveals that randomized studies comparing vaginal cuff closure techniques performed with barbed sutures, Vicryl (polyglactin 910), and PDS (polydioxanone) sutures have not demonstrated any significant differences in adverse events [14,15,16,17]. However, due to the relatively low incidence of vaginal cuff dehiscence, evaluating this complication prospectively poses methodological challenges. Based on these findings, vaginal cuff closure is typically performed using the technique and suture material preferred by the surgeon. The findings of our study are consistent with previous research demonstrating that various intracorporeal suturing methods—such as barbed sutures and continuous Vicryl or PDS sutures—offer comparable safety outcomes for vaginal cuff closure after TLH. While barbed sutures have been shown to reduce suturing time, concerns have been raised regarding their association with increased rates of vaginal cuff dehiscence and bowel complications in some reports [1]. In contrast, AFS provided a reduction in cuff closure time without compromising safety, and without requiring barbed or specialized suture materials. Furthermore, although all surgeries in our study were performed by experienced specialists, the potential influence of a learning curve—especially for novel techniques like AFS—cannot be entirely ruled out. No explicit increase in complications was observed over time. However, future studies might benefit from evaluating the number of cases required to reach procedural proficiency with AFS. This would help define its feasibility for broader surgical adoption and training environments. Taken together, these findings suggest that AFS may serve as a valuable alternative to conventional or barbed suture techniques, particularly in settings where cost control and preservation of pelvic support structures are prioritized.

Although the rate of postoperative vaginal bleeding did not show a statistically significant difference between the groups, it was relatively lower in the AFS group (continuous suturing: five cases vs. AFS: three cases). Additionally, vaginal cuff granulation tissue formation was observed in one patient from each group. However, while one patient in the continuous suturing group required emergency postoperative laparotomy, no such cases were reported in the AFS group. Considering the statistically significant reduction in cuff suturing time, the AFS technique emerges as a strong alternative for vaginal cuff closure, offering potential advantages in surgical efficiency and postoperative outcomes.

Although cases belonging to four surgeons with similar surgical experience and technique were included, the most important limitation of this study is that it was designed retrospectively. However, since it has introduced a new surgical approach to the literature, it will be a pioneer for future prospective studies. Notably, although the retrospective design of the present study may inherently limit the control over potential selection bias, several precautions were taken to ensure methodological rigor. All procedures were performed by experienced gynecologic surgeons who were fully qualified specialists, rather than residents or trainees, thereby ensuring a high level of technical consistency across cases. Furthermore, the choice between AFS and continuous suturing techniques was made without reliance on patient-related criteria, aiming to mitigate allocation bias. While recognizing that randomized controlled trials (RCTs) represent the gold standard for clinical research, our findings serve as a foundation for future prospective investigations designed to validate the clinical utility and long-term safety of the AFS technique in more diverse populations.

Another important limitation of this study is the relatively short follow-up period, limited to 3 and 6 weeks postoperatively. Although early postoperative complications such as bleeding, infection, and vault granulation were systematically assessed, the current study design does not allow for the evaluation of long-term outcomes such as vaginal cuff integrity, pelvic organ prolapse, or sexual function. These outcomes are clinically significant, particularly in procedures aimed at preserving pelvic floor support.

Postoperative complications following total laparoscopic hysterectomy (TLH), though infrequent, may include vaginal cuff dehiscence, surgical site infection (SSI), granulation tissue formation, and urinary incontinence. While our study observed low rates of these events in both groups, the small sample size limits the power to detect rare but clinically important differences.

Recently, there has been growing interest in the use of inflammatory biomarkers to predict postoperative infectious complications. In colorectal surgery, low preoperative levels of butyrylcholinesterase (BuChE) have been associated with an increased risk of surgical site infections and sepsis [18]. Although its role in gynecologic surgery has not yet been clearly defined, BuChE and other novel biomarkers may offer valuable insights for preoperative risk assessment in hysterectomy patients. Further prospective studies are warranted to explore their potential utility in predicting postoperative outcomes in gynecologic procedures.

Future research should include extended follow-up periods with standardized assessments to evaluate the long-term reliability and functional outcomes of the AFS technique. Prospective longitudinal studies are needed to determine whether the early efficiency benefits observed with AFS translate into sustained safety and anatomical stability over time.

In addition, the study did not assess patient-centered outcomes such as postoperative pain, patient satisfaction, or quality of life, which are important measures in evaluating the holistic impact of surgical interventions. Although the present analysis focused on operative efficiency and safety, future studies should incorporate standardized instruments to capture subjective experiences and recovery metrics.

As for cost-effectiveness, both suturing techniques utilized the same suture material (0-Vicryl), and no additional tools or devices were required in the AFS group. Therefore, procedural costs were considered equivalent between the groups under the conditions of this study.

## 5. Conclusions

In conclusion, minimally invasive treatment for hysterectomy has the advantages of less morbidity, shorter hospital stays, and faster return to normal activities, making it popular among surgeons and patients. This situation has brought about the search for safer, more applicable, and practical surgical techniques. This study showed that the AFS technique significantly reduces vaginal cuff closure time. However, the widespread clinical adoption of AFS requires long-term follow-up studies to determine its impact on pelvic floor integrity and the risk of vaginal vault prolapse. Additionally, comparative studies assessing the effects of different vaginal cuff closure techniques on sexual function and patient satisfaction would provide a better understanding of the patient-centered outcomes of this method. Furthermore, prospective studies evaluating the learning curve of AFS among surgeons in training, as well as investigating whether this technique differs from the continuous suturing method in terms of the time required for proficiency, would offer valuable insights into its practical application in surgical training.

## Figures and Tables

**Table 1:** Demographic and Clinical Characteristics of Patients.

Demographic Patient Characteristics	Continue Suture (*n* = 120)	Anchorflow Suture (*n* = 88)	*p*
Mean age, year (SD)	49.5 ± 5.2	50 ± 5.7	0.56
Mean body mass index, kg/m^2^ (SD)	30.5 ± 5	29.3 ± 4.6	0.07
Mean gravida (SD)	3.4 ± 1.8	3.3 ± 2	0.35
Mean parity (SD)	2.8 ± 1.5	2.6 ± 1.5	0.25
Preoperative hematocrit	37.5 ± 4.2	33.2 ± 3.7	0.61
Postoperative hematocrit	37.9 ± 5.7	33.4 ± 3.6	0.35
ASA I, *n* (%)	68 (56.7%)	50 (56.8%)	0.99
ASA II, *n* (%)	52 (43.3%)	38 (43.2%)	0.99
Menopausal situation			
Premenopausal period (*n*)	91	60	0.22
Menopausal period (*n*)	29	28	0.22

**Table 2:** Surgical and Postoperative Outcomes Based on Suture Technique.

	Continue Suture (*n* = 120)	Anchorflow Suture (*n* = 88)	*p*
Hospital stay (d), mean (SD)	2.02 ± 0.5	1.99 ± 0.4	0.84
Length of surgery (min), mean (SD)	103.98 ± 37.5	96.13 ± 10.2	0.15
Time to close vaginal cuff(min), mean (SD)	13.36 ± 2.8	10.26 ± 2.3	<0.001
Preoperative-postoperative hematocrite changes	4.73 ± 2.4	4.49 ± 2.2	0.47
Intraoperative blood transfusion, *n* (%)	0 (0%)	0 (0%)	-

**Table 3:** Comparison of Postoperative Complications.

Postoperative Complications	Continue Suture (*n* = 120)	Anchorflow Suture (*n* = 88)
Emergency relaparotomy	1	0
3-week postoperative findings		
Dehiscence	0	0
Infection		
Bleeding	5	3
6-week postoperative findings		
Vault granulation tissue formation	1	1
Uriner incontinance	2	0

Footnotes: Infection was defined as surgical site infection (SSI), based on clinical findings such as localized pain, redness, purulent discharge, or fever in accordance with CDC criteria.

## Data Availability

Data available in a publicly accessible repository (https://github.com/BerfinUluutku/anchorflow-suture-techniques.git, accessed on: 21 January 2025.
